# Scoping review on prevention of suicidal thoughts and behaviors in adolescents: methods, effectiveness and future directions

**DOI:** 10.3389/frcha.2024.1367075

**Published:** 2024-06-10

**Authors:** Josée Aoun, Michel Spodenkiewicz, Catherine Marimoutou

**Affiliations:** ^1^ CIC-EC 1410, Inserm, CHU de La Réunion, Saint-Pierre, France; ^2^ Moods Team, Inserm UMR-1018, CESP, Le Kremlin-Bicêtre, France; ^3^ McGill Group for Suicide Studies, Department of Psychiatry, Douglas Mental Health University Institute, McGill University, Montréal, QC, Canada

**Keywords:** adolescents, suicide ideation, suicide attempt, suicidal thoughts and behaviors, early intervention, prevention

## Abstract

**Introduction:**

Despite the extensive implementation of suicide prevention strategies targeting suicidal thoughts and behaviors (STB) in adolescents, there remains a concerning lack of improvement in the situation. In this comprehensive scoping review, our objective was to provide insights into prevention methods for suicidal thoughts and behaviors directed towards adolescents, including their effectiveness, public perception, and potential adaptations.

**Method:**

A scoping review was conducted, encompassing 71 articles including systematic review, clinical trials and qualitative studies for a wider understanding. Most articles included focus generally on adolescents aged 10–20.

**Results:**

No single intervention has shown expected effectiveness, collective efforts have laid a solid foundation for suicide prevention. Promising interventions include cognitive-behavioral therapy (CBT) and incorporating Technology-based interventions. However, challenges persist in promoting help-seeking behaviors and addressing barriers such as stigma, the natural impulsive nature of adolescents and difficulty in selecting and defining data and designs.

**Discussion:**

This review underscores the need for a holistic approach to suicide prevention, integrating social, emotional, and psychological dimensions. Successful interventions target underlying issues like depression and loneliness rather than solely focusing on suicidal thoughts and behaviors (STB). Combining direct and indirect interventions is a sensible approach for both immediate and long-term results. Understanding Generation Z's unique needs, influenced by technology and diverse perspectives, is crucial for effective prevention.

**Conclusion:**

Involving adolescents and adopting patient-centered healthcare with outcome measures like Patient Perceived Outcome Measures can enhance suicide prevention efforts by prioritizing safety and patient experiences.

## 1 Introduction

Suicidal thoughts and behaviors (STB) involve thinking about or talking about suicide, making plans to end one's life, attempting suicide, and engaging in self-harm actions that can be fatal (1, 2). Negative self-appraisal stands out as a leading predictor of reasons for dying, emphasizing its notable association with suicidality. Depressed and suicidal individuals commonly exhibit irrational thought patterns characterized by themes of self-depreciation and low self-esteem (3).

The number of youth seeking help for STB through emergency departments, emergency hotlines, aid centers, and similar resources have increased (4–7). People with STB experience ongoing distress, leading to persistent sufferance and frequent utilization of mental health facilities (8). They may not necessarily die of suicide (9), but even professionals are still unable to predict accurately who will (10).

Therefore, the objective of suicide prevention should not only focus on reducing death by suicide, but also on addressing and alleviating suicidal thoughts and behaviors, a primary risk factor for suicide alongside the major risk factors for suicidal thoughts and behaviors (11).

Adolescence is a period of vulnerability that increases the likelihood of developing STB, which can persist into adulthood and become chronic, hindering positive personal development. The adolescence phase is normally characterized by significant changes in the brain and body, including hormonal fluctuations associated with puberty, which impact mood, impulse control, and adequate decision-making abilities (12). Heightened impulsivity, quick mood changes and difficulties rationalizing risks and consequences, are all risk factor that render adolescents more vulnerable to STB (13, 14). There are some physical and biological evidences, on these risks. Brain imaging shows that group differences in surface area in the prefrontal, temporal and parietal regions, as well as in the volume of several subcortical nuclei are associated with the occurrence of suicide attempts in depressed adolescents (12, 15). Additionally, distinct patterns of serotonergic abnormalities, disruptions in the hypothalamic-pituitary-adrenal (HPA) axis, and irregularities in growth hormone (GH) secretion are associated with suicidal behaviors in adolescents (12). Exposure to suicidal behavior, particularly within the family context, and childhood adversities like abuse, neglect, and family conflict also increase the risk of STB (16). The motivation behind suicide attempts among adolescents often relates to interpersonal problems rather than financial or illness-related issues like adults (17).

Traditionally prevention is classified as primary secondary and tertiary but there is a rapid escalation of the classification of universal and selective prevention since suicidal thoughts and behavior like addiction is hard to classify as other disorders as present or absent. Universal and selective prevention are two key approaches in the continuum of prevention strategies (18, 19).

Universal prevention strategies aim to address the entire population's mental health and reduce suicide risk by removing barriers to care and increasing access to support systems. These strategies encompass public education campaigns, school-based programs, means restriction efforts, media education on responsible reporting, and crisis response plans within educational settings. By targeting the entire population rather than just high-risk individuals, universal strategies seek to enhance social support networks and coping skills on a community-wide scale.

In contrast, selective prevention strategies focus on specific high-risk groups within the population, aiming to identify and intervene with vulnerable individuals before suicidal behaviors manifest. These strategies involve screening programs, gatekeeper training for frontline caregivers, support groups tailored to at-risk populations, and improved access to crisis services and treatment. By honing in on factors such as age, sex, occupation, or family history, selective prevention endeavors to prevent the onset of suicidal behaviors among specific subpopulations, thus contributing to a more nuanced and targeted approach to suicide prevention efforts.

This classification can be applied to study feasible prevention methods in young people (20).

The objective of this literature review is to offer a comprehensive overview of prevention methods for suicidal thoughts and behaviors in adolescents. The literature search involves a comprehensive and systematic approach to identify and gather relevant studies to map the existing literature on prevention of suicidal thoughts and behaviors. It includes insights into the nature of prevention methods, their effectiveness, public perception about these methods and possible adaptations.

### 1.1 Identifying the research question

Is there an observable improvement in the effectiveness of both selective and universal prevention methods? Which specific approaches demonstrate significant results, and what are the identified gaps or deficiencies within current prevention methodologies?

## 2 Search method

This scoping follows Arksey and O'Malley's 5 stages of conducting a scoping review (21). A comprehensive literature review was conducted to examine the current knowledge regarding prevention efforts. Relevant articles were identified through searches conducted in databases such as ScienceDirect, PubMed, and Google Scholar. The search query used was {(suicide[Title/Abstract]) AND (prevention[Title/Abstract])) AND (adolescent[MeSH Terms])}. The search was concluded on April 26, 2023, and encompassed a total of 3,493 unique articles.

### 2.1 Identifying relevant studies and study selection

The inclusion criteria focused on

• Articles published after 2010 to ensure a contemporary approach that considers technological and social advancements, while excluding outdated methods.
• Qualitative studies, controlled trials, and systematic reviews.

The exclusion criteria focused on

• Articles of literature and narrative review, as well as editorials and commentaries, were excluded from the analysis because narrative reviews are more likely to include only research selected by the authors, which introduces bias.
• Articles that did not directly address suicide prevention

This broad inclusion is essential for a comprehensive understanding of the complex clinical and social nature of STB in adolescents. Qualitative studies provide rich insights into experiences and perspectives, informing intervention and policy development. Controlled trials evaluate the effectiveness of interventions, while systematic reviews synthesize evidence for reliable conclusions. This holistic approach enhances understanding, validity, and evidence-based decision-making. This type of rapid review might not describe research findings in any detail but is a useful way of mapping fields of study where it is difficult to visualise the range of material that might be available.

**Figure 1:**
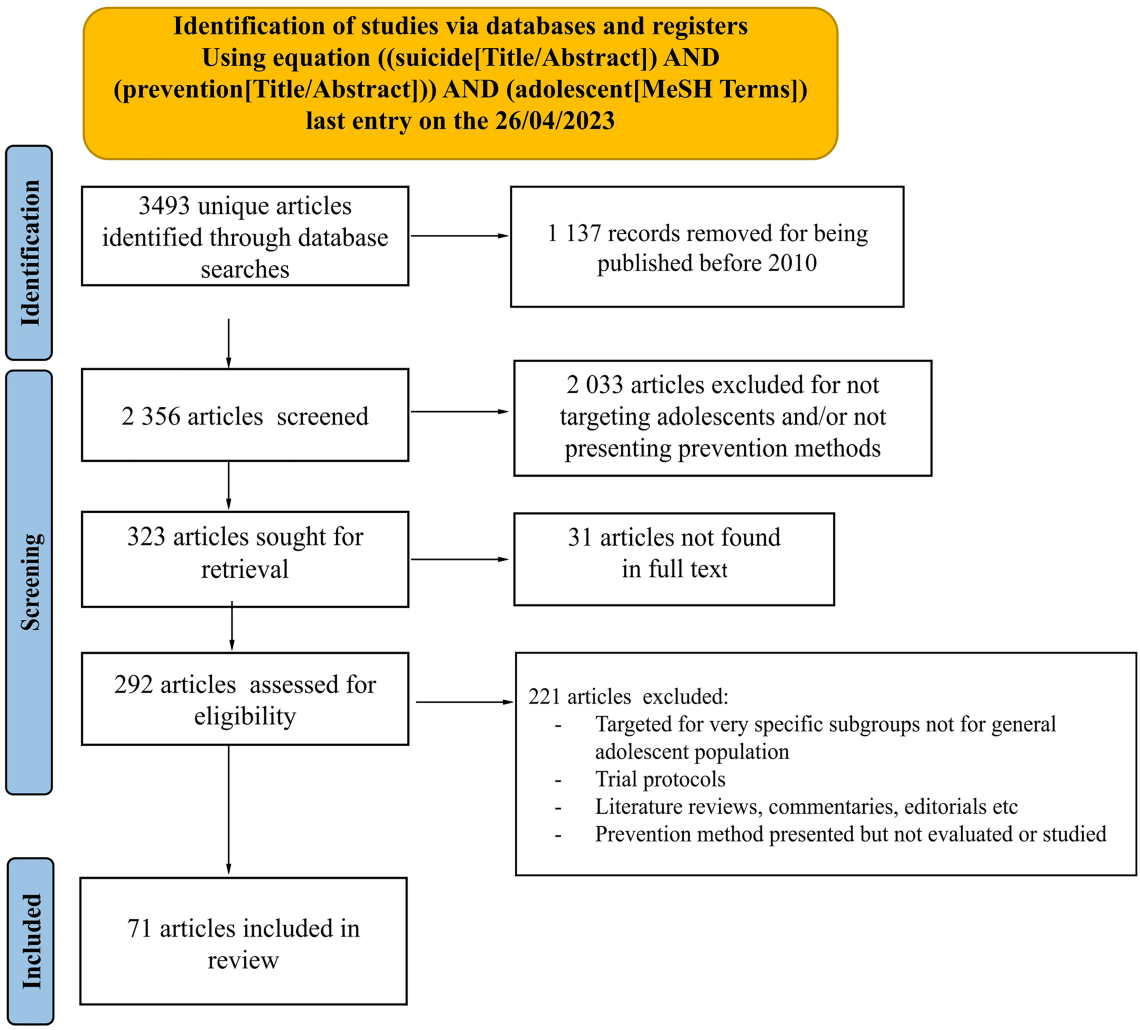
Prisma flowchart: identification and inclusion process.

This scoping review was conducted according to the Preferred Reporting Items for Systematic reviews and Meta-Analyses extension for Scoping Reviews (PRISMA-ScR) Statement [Supplementary Material]. The screening and inclusion process is presented Figure 1.

We sequentially evaluated the titles, abstracts and then full text of all publications identified by our searches for potentially relevant publications. Disagreements were solved by consensus between the reviewers. Descriptive data were manually extracted from eligible articles. To avoid any potential misinterpretation or misrepresentation of the original findings, we quoted findings with minimal syntax modifications to ensure accuracy when conveying results of included articles. Quoted outcomes have been classified in Supplementary Table S1 detailing the author's interpretations and satisfaction of their results.

## 3 Results

All included articles and their main results have been grouped in the table.

In this review, we included 10 systematic reviews (22–31) and 1 meta-analysis (32) encompassing 318 studies. Despite the large number of studies included, several systematic reviews identified limited evidence regarding effective interventions for preventing adolescent suicide. The findings from these reviews were generally underwhelming, as 10 out of 11 of them revealed minimal to no effect in most studies. However, it is worth noting that the studies focusing on cognitive-behavioral therapy (CBT) methods showed some promising outcomes.

### 3.1 On selective prevention

#### 3.1.1 Cognitive-behavioral therapy (CBT)

Has demonstrated effectiveness as a preventive measure against suicide. In this review, we included seven studies that explored various applications of CBT, such as BriefCBT (33), MoodRegulation-CBT (34), the adaptation of CBT into a mobile app called MEMO (35), and programs like CATCH-IT (36, 37) and EMPATHY (38). Notably, the CATCH-IT program showed significant result satisfaction. These interventions effectively target the vulnerabilities of hopelessness and difficulties in coping, which are associated with suicidal behavior. Additionally, dialectical behaviors therapy (DBT) (39, 40) has shown efficacy in preventing suicide attempts. However, the scalability of DBT and individual psychodynamic psychotherapies limits their effectiveness on a larger scale.

#### 3.1.2 Medication and hospitalization

A study examined the frequency of lithium, clozapine, and electroconvulsive therapy (ECT) use and its correlation with suicide deaths. The findings indicated an inverse correlation between the prevalence of suicide and the frequency of using these medications, but this effect was observed mainly among male adolescents (41). While hospitalization is considered a reliable means of ensuring the safety of adolescents, both staff and patients have negative experiences (42–44), particularly in the emergency department, according to surveys and interviews, details of the interviews include reports of hospital staff displaying rudeness towards adolescents and expressing opinions that these individuals have no business being in an emergency department setting. Furthermore, participants highlighted excruciatingly long waiting times, with some indicating that the experience exacerbated their already intense subjective feelings of (STB). Some adolescents even reported being reluctant to seek help again due to their negative encounters, obstructing the promotion of help-seeking behaviors.

#### 3.1.3 Gatekeeper programs

Due to scalability concerns, community programs offer a more viable approach. We have included multiple community and caregiver-based programs that have demonstrated significant outcomes. The primary objective of these programs is to enhance knowledge about appropriate responses and actions when faced with someone at risk of suicide, while simultaneously addressing barriers to accessing care. For example, the P and C CARE programs (45, 46), Connect program (47), and the Family Bereavement Program (FBP) have exhibited notable effectiveness, with the latter showing long-lasting effects on STB even up to a 15-year follow-up period for FBP in one study (48). Additionally, various community education programs have been implemented, specifically targeting different types of caregivers to increase their knowledge and risk management skills.

#### 3.1.4 Coping skills program

Programs that are addressed to adolescents who have STB, such as Surviving the Teens (49, 50), Sources of Strength (51, 52), The Listening Guide (53), and Youth Awareness of Mental Health (54), share a common objective of providing adolescents with essential coping skills and risk management strategies.

#### 3.1.5 Interventions on depression and ill being

In addition, numerous interventions originally developed to target depression or general states of ill-being, have been found to be effective in reducing suicidal thoughts and behaviors (STB), as indicated by various studies included in this review (Supplementary Table S1). The one meta-analysis specifically highlighted a multivariate meta-regression analysis that indicated that studies specifically aimed at targeting STBs had a significantly lower effect size for suicide attempts (32). Therefore, direct methods of prevention of STB appear less effective than indirect methods that target risk factors of STB such as ill being and Depression.

### 3.2 On universal scale prevention

Government programs have also played a significant role in addressing suicide prevention, with, for example, the Garret Lee Smith (GLS) memorial program emerging as a prominent example in the US (55, 56). This program involves community-wide efforts, funding for research, and the development of tools to combat suicide. According to the authors, over a 7-year period, the absence of the program would have resulted in 13.3 more deaths per 100,000 youths (55). Simultaneously, another study showed there were no significant difference in terms of adult suicide mortality rates the year after the implementation, making it specific for adolescents (56).

#### 3.2.1 Community principles

A qualitative study included in this review examined how monks in Thailand adhere to doctrines for well-being and are generally joyful, with Buddhism playing a role (57). Their beliefs align with many suicide prevention principles such as knowledge of life after death, early identification of mental disorders and help seeking, and measures to control suicide methods, alcohol, and drug abuse.

### 3.3 Help seeking methods

Despite the implementation of numerous strategies and programs, the majority of studies have revealed that help-seeking behaviors among adolescents has not increased significantly (Supplementary Table S1). Various barriers contribute to this reluctance, including the stigma surrounding mental illness halting help seeking (22, 44, 47, 58, 59). Additionally, a lack of knowledge about available resources and how to access them poses a significant obstacle and is where gatekeeper programs could come in handy. Moreover, a lack of trust in mental health professionals or discomfort in discussing personal problems with them hinders help-seeking efforts (42, 43, 59–62). A panel of experts participated in a Delphi survey included in this review, which aimed to explore the efficacy of suicide prevention programs. Although it was emphasized that these programs should be tailored and adapted, experts identified that providing information about where to seek help was the most effective component of these programs (28). With regard to help seeking, they suggested that adolescents often confide in their friends, indicating the importance of gatekeeper programs targeting fellow adolescents to provide support and encourage assistance-seeking (28, 62).

Adolescents show overall satisfaction with suicide prevention programs and demonstrate willingness to actively contribute ideas for suicide prevention efforts. Parents, especially mothers, expressed interest in becoming essential allies in suicide prevention, as their engagement has proven to be of significant importance in addressing this issue (40, 45). In most studies, parental presence seems to be a driving factor for suicide prevention, with one study consisting of interviews of young people provided that parents and teachers were the most influential for adolescents compared to other actors like counselors or public workers (63).

### 3.4 Use of technology

A common theme is also the use of technology based prevention. Mobile-based technologies have demonstrated effectiveness in assessing and managing risk among vulnerable young people. Mobile apps provide increased accessibility to therapeutic interventions, particularly for at-risk individuals who may not actively seek help. They offer the opportunity for evidence-based interventions to be accessed multiple times a day, precisely when needed the most. Some examples of technology-based interventions for a selective prevention of suicide included in this review are the #chatsafeguidelines (64, 65), CalmHarm app (66), MEMO (35), and SafePlan app (67), all showed promising feasibility acceptance. Moreover, teenagers often confide in their friends, utilizing social platforms such as Instagram, including features like *close friends’* stories (62), to seek support and share their experiences. These platforms can also play a role in improving help-seeking behavior. The findings extracted from the remainder of included articles (67–92) are synthesized and presented in Supplementary Table 1.

## 4 Discussion

The purpose of this review is to provide a comprehensive understanding of suicide prevention. Descriptive data were manually extracted from eligible articles by directly quoting the findings from clinical trials, expert analyses from systematic reviews, and the direct citations of adolescents and the general public regarding prevention strategies. While some results of suicide prevention interventions have been deemed unsatisfactory by critics and the authors themselves, this only highlights the complex nature of STB. We also observed that the most challenging aspect of suicide prevention lies in identifying and reaching out to those who suffer from STB and encouraging them to seek the necessary help.

This task becomes particularly intricate when dealing with adolescents, as they may not explicitly express a desire to die but exhibit behaviors and communication that contribute to their own depreciation. We found that help-seeking behaviors is halted by the stigma and past negative experiences, and these feelings can be partially alleviated when parents become supportive allies. Additionally, technology and its scalability factor can play a facilitative role. Therapies, which have proved to be effective, such as Cognitive Behavioral Therapy (CBT), can be adapted in the development of community programs, online guidelines, and mobile apps aimed at encouraging adolescents to seek help before self-harm occurs.

Participants of the different included studies seemed to be interested in being active members in modulating and adapting prevention methods. Surveys and qualitative studies have revealed that adolescents provide valuable insights and ideas, even when not explicitly solicited for solutions. All groups of participants (parents, counselors, mental health care professionals and adolescents) have expressed satisfaction and willingness to engage in prevention programs, despite some quantitative results falling short of expectations, highlighting a potential gap in proving method effectiveness, notably for help seeking behavior. While some critics undermine the impact of prevention programs and whether they should keep receiving funding, no alternative or specific program has emerged as the definitive solution. Even though intervention programs may not have demonstrated ground-breaking results, they still play a crucial role in initiating conversations about suicide and gradually dismantling the associated stigma (93–95), a major barrier to seeking help.

While opinions differ on how best to prevent STB, the existing research suggests that interventions targeting underlying issues like depression, loneliness, and hopelessness have been successful in indirectly reducing STB. One of the pillars of suicide prevention according to the WHO website is “*foster socio-emotional life skills in adolescents*”, this approach considers that adolescents needs help in developing skills that allow them to manage emotions, develop a sense of identity and empathy (96–98). STB is a symptom of deeper suffering with the root cause, general ill-being, targeted through interventions. Therefore, we emphasize the importance of treating the root causes using methods not directed towards STB, but towards underlying causes, for long-term results. In contrast, direct interventions on STB tended to have more immediate effects, which in times of crisis or extreme risks for a suicide attempt, could be very valuable (99). It could encourage patients to stay hopeful in their treatment alongside healthy momentary sufferance relief. Combining both direct and indirect interventions could be a sensible approach.

### 4.1 Study limitations

There were some questions raised when going through all the studies included in this review. One was the lack of detailed information about the interventions or programs being studied, especially regarding the items included in prevention programs. In order to gain a better understanding, a basic online search had to be made to visit the websites of these interventions while being mindful that websites could be biased as it was presented by the creators of the programs. Another bias was found in studies funded by program contributors.

The challenges in suicide prevention encompass two main aspects: inadequate availability of data (100) and insufficient research design (101, 102). These limitations impede our ability to accurately estimate suicide rates, evaluate the effectiveness of prevention programs and assess the impact of interventions. To overcome these challenges, it is crucial to enhance data collection and evaluation processes, taking into consideration that STB and suicide itself are not registered in a systematic uniform way worldwide. If the same coding system was implemented, this could enable us to effectively monitor the success of prevention interventions and closely track cases of hospitalized suicide attempts. The 10th revision of the International Statistical Classification of Diseases and Related Health Problems (103) identifies some elements of self-harm and also suicide attempts with a code classification, however the broad nature of suicidal thoughts and behaviors makes their classification complicated since different schools of thoughts have different agreement to what is considered suicide behavior (104, 105).

Furthermore, our research indicated a limited mention on the role of bullying in STB among adolescents. Despite bullying being associated with STB reported in emergency department (106), it was not generally addressed in the interventions and programs we reviewed. Especially noting that any participation in bullying is linked to STB in adolescnets (107).

This study has several limitations. The search terms use while extensive are not exhaustive and could have omitted multiple studies with concrete effective prevention methods. Selecting comprehensive search terms can be challenging, especially when targeted prevention for teens is relatively limited compared to adults. This study solely relied on excerpts from the included articles. This descriptive approach may lack the depth and rigor of an analytical study, potentially leading to oversimplification of complex data. By solely relying on quoted findings, there is a risk of bias as the interpretation of results may be influenced by the authors of the included studies. This review focused on finding gaps in prevention method studies directed towards adolescents. Many included studies focused on Europe and North America and East Asia limiting our understanding of prevention studies in other parts of the world.

### 4.2 Perspectives: understanding Generation Z perspective to adapt prevention strategies

Generation Z or Gen Z for short, born between the mid-to-late 1990s and the early 2010s experiencing a dynamic demographic landscape (108). Renowned for being digital natives, they are the first cohort to grow up with widespread access to technology and the internet, displaying impressive proficiency in utilizing various technological platforms and social media. Additionally, Gen Z is widely recognized for their social-mindedness and progressive outlook, actively advocating for issues such as climate change, social justice, and equality. They demand purpose and accountability from institutions, tirelessly striving for greater equity and sustainability (108, 109).

When considering suicide prevention methods for adolescents, it is important to note that generations do not have the same adolescence, each generation has its particularities. Millennials who experienced their adolescence from mid 1980s to mid 1990s cannot be compared to the adolescence of our current young generation GenZ since the socio-cultural and economic situation of the globe is now vastly different. In addition, our scientific understanding of adolescence has seen major improvements, and we now know that adolescents are in a distinct stage of development that differs greatly from adulthood.

Although we are gradually improving destigmatizing mental health problems and encouraging expression, we are often reluctant to listen to and understand adolescents when they confide in us, and to take rational steps to direct them to the help they need. Understanding the unique needs of adolescents, fostering social support, and providing constructive guidance are crucial.

Care approaches that integrate social aspects are proving more effective than medical treatment alone (110). This holistic approach considers the adolescent in his or her entirety, considering cultural, social, and psychological dimensions, with the aim of promoting well-being. Interventions are focusing mainly on listening, patience and empowerment, so as to prepare the teenager to face their own difficulties (111). The aim is to provide them with the resources they need to develop their skills and find the answers specific to them by themselves. Prevention efforts focus on promoting healthy coping mechanisms, rational thinking, and a positive mindset, not only for suicidal adolescents but for their whole entourage. Involving adolescents in both prevention plans and prevention initiatives utilizing technology could maximize effectiveness.

Healthcare primarily focuses on reducing symptoms, minimizing disability, and enhancing the quality of life, which are aspects that can only be adequately assessed by patients themselves. Patients genuinely appreciate being involved in their healthcare decisions, and this active participation can lead to additional health benefits.

Using patient-reported outcome measures (PROMs) helps to avoid observer bias, which could be inevitable if clinicians were to assess their own practice (112). By considering patients’ perspectives and experiences, the process enhances the public accountability of health services and healthcare professionals, leading to a more patient-centred and transparent healthcare system (113, 114).

## 5 Conclusion

The efforts aimed at preventing suicide have significantly increased, initiating conversations and challenging the stigma surrounding STB. Although no single approach has shown outstanding efficacy, these endeavors have laid a solid foundation for future progress. Technological advancements provide a promising avenue to address scalability issues and enhance help-seeking processes, offering autonomy and anonymity. However, there is a need to adapt these methods to different socio-economic contexts, while also considering the unique challenges faced by new generations. To achieve meaningful impact, ongoing work is required to ensure that prevention strategies align with modern issues and cater to diverse populations. Combining technological innovations with a comprehensive understanding of societal factors, can allow continuing suicide prevention efforts and foster more support and resilience.
