# Supplementary material for: Scoping review on prevention of suicidal thoughts and behaviors in adolescents: methods, effectiveness and future directions
Source: Front Child Adolesc Psychiatry. 2024 Jun 10;3:1367075. doi: 10.3389/frcha.2024.1367075 (PMC11748894; doi:10.3389/frcha.2024.1367075)
Supplement: Supplementary file 2 [file Table1.pdf]

Reference citation number in text	Citation of article	Population and Participants	Study Design	Targeted intervention and discription	Main quoted outcomes
22	Torok M, Calear AL, Smart A, Nicolopoulos A, Wong Q. Preventing adolescent suicide: A systematic review of the effectiveness and change mechanisms of suicide prevention gatekeeping training programs for teachers and parents. Journal of adolescence. 2019 Jun 1;73:100-12.	13 studies	Systematic Review	Assessing knowledge(litteracy), confidence(self efficacy), attitude(context of stigma), gatekeeper behavior(the identification of at risk youth) through litterature review	- No significant difference could be found between teachers and parents in the effectiveness of gatekeeper studies<br>- Participants showed improvement in knowledge and perceived confidence to intervene.<br>- However, no improvement was observed in behavioral outcomes, indicating that skill-based interventions might be necessary.<br>- The findings suggest that the specific type of program used may not be as crucial as ensuring that some form of suicide prevention gatekeeper training is implemented among key gatekeeper groups.
23	Doty B, Bass J, Ryan T, Zhang A, Wilcox H. Systematic review of suicide prevention studies with data on youth and young adults living in low-income and middle-income countries. BMJ open. 2022 Sep 1;12(9):e055000.	44 elligible studies	Systematic Review	- psychological intervention<br>- public awareness campaign<br>- biomedical treatments<br>- restriction means<br>- multiple interventions	- There is a lack of intervention studies addressing youth and young adults in low-income and middle-income countries (LMIC) regarding suicide, despite it being a significant public health issue in these countries.<br>- The available number of intervention studies does not adequately match the magnitude of the problem
24	Cox G, Hetrick S. Psychosocial interventions for self-harm, suicidal ideation and suicide attempt in children and young people: What? How? Who? and Where?. BMJ Ment Health. 2017 May 1;20(2):35-40.	44 articles included	Systematic Review	review of psyhcosocial interventions with a focus on CBT types. Possible inovation of this intervention to better adapt to recent internet technologies	Trials using Cognitive Behavioral Therapy (CBT) targeting self-harm have shown promising results.<br>Other methods have not shown significant results in reducing suicidal ideation (SI), self-harm (SH), or suicide attempts (SA)
25	Devenish B, Berk L, Lewis AJ. The treatment of suicidality in adolescents by psychosocial interventions for depression: A systematic literature review. Australian & New Zealand Journal of Psychiatry. 2016 Aug;50(8):726-40.	35 articles	Systematic Review	A systematic review of psychological interventions aimed to prevent and/or treat depression in adolescents in which outcomes for suicidality were reported	- Most structured psychological depression treatment interventions did not show superior outcomes compared to pharmaceutical or treatment as usual control groups.<br>- Depression prevention studies showed small but statistically significant reductions in suicidality.<br>- Cognitive Behavioral Therapy (CBT) interventions resulted in reductions in suicidality with moderate effect sizes, and they were at least as effective as pharmacotherapy in reducing suicidality. However, it remains unclear whether these effects are maintained over time
26	Szlyk H, Tan J. The role of technology and the continuum of care for youth suicidality: systematic review. Journal of medical internet research. 2020 Oct 9;22(10):e18672.	26 studies	Systematic Review	multiple technology based intervention (multiple level intervention) examinening the efficacy of technology-enhanced youth suicide prevention and interventions across the continuum of care.	- Few studies in the reviewed sample utilized technology for assessing suicidality or initiating mental health treatment.<br>- In the suicidology literature, there is a lack of specific information about the technological components of interventions, with a focus on evaluating the intervention as a whole.<br>- Many studies, regardless of their design, share common underdeveloped domains in the Measurement of Quality of Research Studies (MQRS)

Reference citation number in text	Citation of article	Population and Participants	Study Design	Targeted intervention and discription	Main quoted outcomes
27	Morken IS, Dahlgren A, Lunde I, Toven S. The effects of interventions preventing self-harm and suicide in children and adolescents: an overview of systematic reviews. F1000Research. 2019;8.	8 articles	Systematic Review	Evaluate the effects of interventions preventing self-harm and suicide in children and adolescents in an overview of systematic reviews. Comparison intervention versus TAU or no intervention	It is important to note that the quality of evidence supporting these interventions is generally considered to be of "very low" or "low" quality. This grading reflects the overall confidence in the accuracy of the effect estimates, rather than the quality of individual studies.
28	Grosselli L, Herzog K, Aseltine RH, Balazs J, Carli V, Ciffone J, De Leo D, Van der Feltz-Cornelis C, Hawton K, Hegerl U, Kõlves K. Dos and don'ts in designing school-based awareness programs for suicide prevention: Results of a three-stage Delphi survey. Crisis: The Journal of Crisis Intervention and Suicide Prevention. 2021 May 27.	23 experts	Delphi Survey	evaluate current prevention methods	- Although participants argued in favor of school-based awareness programs, they highlighted the need for both adapting these programs to the audience and implementing plans to prevent adverse effects<br>- Only one item was rated as very effective for suicide prevention namely, "Information about where to find help (in the community, at school).<br>- Teenagers often disclose suicidal thoughts to their friends
29	Forte A, Sarli G, Polidori L, Lester D, Pompili M. The role of new technologies to prevent suicide in adolescence: a systematic review of the literature. Medicina. 2021 Jan 26;57(2):109.	12 studies	Systematic Review	Provide an overview of the present literature on the use of new technologies in adolescent suicide prevention.	New technologies were found to be well accepted and tolerated supports for suicide prevention in adolescents. However, to date, few data support the use of such interventions in clinical practice and preventive strategies
30	Aguirre Velasco A, Cruz IS, Billings J, Jimenez M, Rowe S. What are the barriers, facilitators and interventions targeting help-seeking behaviours for common mental health problems in adolescents? A systematic review. BMC psychiatry. 2020 Dec;20(1):1-22.	90 studies	Systematic Review	Understanding help-seeking behaviors in adolescents who are most in need of assistance.	- The quality of studies was low to medium and there was no general agreement regarding help-seeking definition and measurements.
31	Gijzen MW, Rasing SP, Creemers DH, Engels RC, Smit F. Effectiveness of school-based preventive programs in suicidal thoughts and behaviors: A meta-analysis. Journal of affective disorders. 2022 Feb 1;298:408-20.	11 studies	Meta Analysis	Programs that target known risk factors for STB such as depression	- The studies are not similar enough to be quantitatively synthesized, the meta-analysis which was based on posttest assessments showed a substantial amount of heterogeneity<br>- Multivariate meta-regression analyses indicated that studies that were specifically aimed at targeting STBs had a significantly lower effect size for SA<br>- no conclusion on intervention indirect to STB, only one study found
32	Sinyor M, Williams M, Mitchell R, Zaheer R, Bryan CJ, Schaffer A, Westreich N, Ellis J, Goldstein BI, Cheung AH, Selchen S. Cognitive behavioral therapy for suicide prevention in youth admitted to hospital following an episode of self-harm: A pilot randomized controlled trial. Journal of affective disorders. 2020 Apr 1;266:686-94.	21 patients analyzed with many had just been admitted for suicide attempt	RCT	Brief CBT for Self Harm, a maladaptive form of coping that is the primary target of treatment and usual care of attentional control tratment	The frequency of repeat self-harm (SH) during acute treatment was statistically significantly lower in the BCBT (Brief Cognitive-Behavioral Therapy) group

Reference citation number in text	Citation of article	Population and Participants	Study Design	Targeted intervention and discription	Main quoted outcomes
33	Högberg G, Hällström T. Mood regulation focused CBT based on memory reconsolidation, reduced suicidal ideation and depression in youth in a randomised controlled study. International Journal of Environmental Research and Public Health. 2018 May;15(5):921.	15 MR -CBT, 12 TAU	RCT	MR-CBT focuses on mood regulation by means of counter conditioning with memory reconsolidation .<br>Subjects practice keeping emotionally positive memories to diminish the emotional impact of negative memories.<br>The program is based on the principles of cognitive-behavioral therapy (CBT) and aims to help individuals develop skills to manage their emotions and thoughts	- Suicidal events were significantly reduced in the MR-CBT group at the end of treatment, whereas no significant reduction was observed in the TAU<br>- Both treatment groups showed significant improvements in depression and overall well-being.<br>- While the findings are not definitive, they provide encouraging evidence for the effectiveness of MR-CBT in reducing suicidal events and improving mental health outcomes
34	Whittaker R, Merry S, Stasiak K, McDowell H, Doherty I, Shepherd M, Dorey E, Parag V, Ameratunga S, Rodgers A. MEMO—a mobile phone depression prevention intervention for adolescents: development process and postprogram findings on acceptability from a randomized controlled trial. Journal of medical Internet research. 2012 Jan 24;14(1):e1857.	1348 students 13–17 yo, 855 were eventually randomly assigned to groups	RCT	MEMO is a mobile intervention in the form of 15 key messages derived from cognitive behavioral therapy (CBT).<br>The program was fully automated and delivered in 2 mobile phone messages/day for 9 weeks, with a mixture of text, video, and cartoon messages and a mobile website.<br>Delivery modalities were guided by social cognitive theory and marketing principles<br>The intervention was compared with an attention control program of the same number and types of messages on different topics	- Participants in the intervention group reported that the intervention helped them become more positive (66.7% or 279 out of 418 participants) and eliminate negative thoughts (50.2% or 210 out of 418 participants).<br>- The proportions of participants experiencing these positive effects were significantly higher in the intervention group compared to the control group
35	Dickter B, Bunge EL, Brown LM, Leykin Y, Soares EE, Van Voorhees B, Marko-Holguin M, Gladstone TR. Impact of an online depression prevention intervention on suicide risk factors for adolescents and young adults. Mhealth. 2019;5.	youth aged 14 to 24	Pre and post evaluation	Competent Adulthood Transition with Cognitive behavioral Humanistic and Interpersonal Training (CATCH-IT)<br>was designed to teach coping skills to teenagers and young adults.<br>14 self-guided, online modules that use techniques from cognitive-behavioral therapy (CBT)<br>Compared baseline and postvention but also completers and non completers	- The mean suicidal ideation across all participants decreased by 3.3% from baseline.<br>- Among those who completed all 14 modules (n=24), the mean suicidal ideation decreased by 8.8%.<br>- Changes in suicidal ideation were associated with changes in low self-esteem, but not with hopelessness and social isolation.<br>- For non-completers of the CATCH-IT program, the effect size was small.
36	Soares EE, Giordano BL, Rogers J, Leykin Y, Cordova M, Van Voorhees B, Gladstone TG, Bunge EE. The effects of engagement with an online depression prevention program for adolescents on suicide risk factors. Journal of technology in behavioral science. 2022 Sep;7(3):307-14.	369 adolescents aged 13–18 with a type pf depression diagnosis	RCT	comparing the CATCH it and Health education modules	- The present analyses support the roles of social isolation and social rejection in hopelessness, which is in turn closely linked to suicidal ideation; the relationship between social isolation and rejection and hopelessness replicates existing literature<br>- More engagment in the program increased the significance of the effect of the porgram on decreasing suicide risk factors

Reference citation number in text	Citation of article	Population and Participants	Study Design	Targeted intervention and discription	Main quoted outcomes
37	Silverstone PH, Bercov M, Suen VY, Allen A, Cribben I, Goodrick J, Henry S, Pryce C, Langstraat P, Rittenbach K, Chakraborty S. Long-term results from the empowering a multimodal pathway toward healthy youth program, a multimodal school-based approach, show marked reductions in suicidality, depression, and anxiety in 6,227 students in grades 6–12 (aged 11–18). Frontiers in psychiatry. 2017 May 15;8:81.	6,227 students who were assessed at least once during the study period.	Prospective Cohort Study	Empowering a Multimodal Pathway Toward Healthy Youth (EMPATHY).<br>The EMPATHY multimodal program consisted of repeated data collection, identification of a high-risk group, a rapid intervention for this high-risk group including offering supervised online cognitive behavioral therapy (CBT) program<br>The program is multimodal and consists of the following components:<br>- Repeated data collection<br>- Identification of a high-risk group<br>- A rapid intervention for this high-risk group including offering supervised online cognitive-behavioral therapy (CBT) program<br>- A universal CBT intervention for those in Grades 6-8<br>- A variety of interactions with trained staff ("Resiliency Coaches")<br>- Referral to external medical and psychiatric services where appropriate	- Highly statistically significant decreases in suicidality rates<br>- There were also highly statistically significant reductions in depression and anxiety scores at each time-point.
38	Kennard B, Mayes T, King J, Moorehead A, Wolfe K, Hughes J, Castillo B, Smith M, Matney J, Oscarson B, Stewart S. The development and feasibility outcomes of a youth suicide prevention intensive outpatient program. Journal of Adolescent Health. 2019 Mar 1;64(3):362-9.	415 Patients (ages 12–18) with a recent suicide attempt or worsening of suicidal ideation	Pre and post evaluation + follow up	intensive outpatient program (IOP)<br>intervention approach is primarily Cognitive Behavior Therapy (CBT)<br>it includes components of Dialectical Behavior Therapy, mindfulness CBT and Relapse Prevention CBT.<br>The IOP included 3 hours of group therapy twice weekly for 4–6 weeks, depending on individual need.<br>Treatment also included individual and family therapy, medication management as needed, and a one-hour weekly skills-based parent psychoeducation group	- At discharge, both parents and patients reported a significant improvement in depression severity based on the QIDSA compared to baseline<br>- Scores on the CHRT were significantly reduced at discharge with propensity scores and risk scores demonstrating a marked improvement.
39	Babeva KN, Klomhaus AM, Sugar CA, Fitzpatrick O, Asarnow JR. Adolescent suicide attempt prevention: Predictors of response to a cognitive–behavioral family and youth centered intervention. Suicide and Life-Threatening Behavior. 2020 Feb;50(1):56-71.	50 youths drawn from two previously published trials:	Pre and Post evaluation	Safe Alternatives for Teens and Youth (SAFETY) a time-limited, DBT-informed cognitive-behavioral treatment,<br>designed to address challenges in treating adolescents with SA/SH.<br>The program is family-oriented and lasts for 12 weeks<br>The goal of the program is to reduce suicide risk by improving problem-solving skills, encouraging family support, and increasing safety	- This study provides data from two samples indicating medium-to-large effect sizes for the SAFETY treatment on measures of youth SB, depression, hopelessness, and social adjustment in the domains of school, home, and with peers, as well as a large effect size for parental depression.
40	Desai Boström AE, Andersson P, Rask-Andersen M, Jarbin H, Lundberg J, Jokinen J. Regional clozapine, ECT and lithium usage inversely associated with excess suicide rates in male adolescents. Nature communications. 2023 Mar 14;14(1):1281.	male adolescents	Correlational research	Evaluate the evolution in suicide rates with the introduction of regional clozapine, ECT and lithium usage frequencies in 15–19-yo male adolescents	This study demonstrates that regional clozapine, ECT and lithium usage frequencies in 15–19-year-olds are associated with reductions in excess regional suicide death rates in male adolescents
41	Byrne SJ, Bellairs-Walsh I, Rice SM, Bendall S, Lamblin M, Boubis E, McGregor B, O'Keefe M, Robinson J. A qualitative account of young people's experiences seeking care from emergency departments for self-harm. International journal of environmental research and public health. 2021 Mar 12;18(6):2892.	13 young people, 17–25 years with female majority	Exploratory mixed-methods	Collecting information on the Emergency Department experience and determining the optimal time and approach to engage with young people who have previously sought help for suicidal tendencies after they have been discharged from the ED	- The negative feelings towards themselves, including anger and shame, were subsequently exacerbated among participants, impacting their presentations in the emergency department. Many expressed a sense of embarrassment.<br>- The lack of privacy affected the amount of information disclosed by participants to staff.<br>- One participant recalled attempting self-harm when unsupervised.<br>- Staff were perceived as disinterested, dismissive, and lacking in knowledge.

Reference citation number in text	Citation of article	Population and Participants	Study Design	Targeted intervention and discription	Main quoted outcomes
42	Freeman J, Strauss P, Hamilton S, Pugh C, Browne K, Caren S, Harris C, Millett L, Smith W, Lin A. They Told Me "This Isn'ta Hotel": Young People's Experiences and Perceptions of Care When Presenting to the Emergency Department with Suicide-Related Behaviour. International journal of environmental research and public health. 2022 Jan 26;19(3):1377.	35 young people aged 16–25 years	Inductive approach based on focus group interview analysis	Emergency Department dealing with Suicide-Related Behaviour	- Young people in the study described the emergency department (ED) as their only option for help with suicidal thoughts and behaviors, despite previous negative encounters.<br>- Similar findings have been reported in other studies, where the ED is seen as the only available choice for individuals with various mental health concerns.<br>- Young people recounted not being taken seriously in the ED and experiencing lengthy wait times, sometimes up to 15 hours, before receiving appropriate care
43	Libon J, Alganion J, Hilario C. Youth perspectives on barriers and opportunities for the development of a peer support model to promote mental health and prevent suicide. Western journal of nursing research. 2023 Mar;45(3):208-14.	11 participants aged 15–24.	Qualitative descriptive approach	qualitative descriptive approach to investigate the nuances of lived experiences, perspectives, and systems influencing youth mental health needs and access to services	- Financial inequities, including the cost of mental health services and transportationt. Participants expressed frustration with the slow progress in government support.<br>- Communicating with others and coping with stressors became significant challenges for participants since the COVID-19 outbreak.<br>-.Participants noticed a greater public discourse and reduced stigma surrounding mental health.<br>- Connecting with others in the community was highlighted as an important aspect of mental health care and should be integrated into mental health initiatives.<br>- Social media was identified as a valuable platform for sharing experiences and connecting with others to support mental health
44	Hooven C. P arents-CARE: A Suicide Prevention Program for Parents of At-Risk Youth. Journal of child and adolescent psychiatric nursing. 2013 Feb;26(1):85-95.	289 housholds (kids and their parents for a total of about 600 poeple)	RCT	Acceptability and feasability of Parents-Care program:<br>a brief youth suicide prevention program for parents, targeting family communication.<br>is a focused intervention that relies on a theoretical framework comprised of aspects of social learning theory, motivational interviewing, social support, and skills acquisition theories to promote change in parent–teen communication	- Feasibility: Approximately 20% of households had both parents participating in the program, while the majority of participants were mainly mothers. Father participation was less than 10%.<br>- Youth participation in the program was high, indicating their engagement and commitment to the program.<br>- Parents demonstrated acceptance of the program, and a follow-up conducted after 2 months revealed that parents continued to utilize the skills they learned in parenting, including providing support and actively listening to their children.
45	Hooven C, Walsh E, Pike KC, Herting JR. Promoting CARE: including parents in youth suicide prevention. Family & community health. 2012 Jul;35(3):225.	A total of 615 high school youth and their parents participated.	RCT	The C-CARE intervention—C-CARE is an assessment and intervention for youth that addresses multiple, co-occurring risk factors related to youth suicide and has been shown to reduce suicidal behaviors, emotional distress, and alcohol and drug use<br>It uses social learning, motivation, social support, and skills-acquisition theories to promote behavioral change relative to the targeted risk factors for youth suicide<br>.The P-CARE intervention—P-CARE is a suicide prevention "first aid" and skillstraining intervention for parents, designed to complement and augment the behavior change that is targeted in C-CARE by targeting family processes implicated in youth suicide risk (eg, conflict, lack of support).	Results reveal that the youth intervention and combined youth and parent intervention produced significantly greater reductions in suicide risk factors and increases in protective factors

Reference citation number in text	Citation of article	Population and Participants	Study Design	Targeted intervention and discription	Main quoted outcomes
46	Bean G, Baber KM. Connect: An effective community-based youth suicide prevention program. Suicide and Life-Threatening Behavior. 2011 Feb;41(1):87-97.	648 adults and 204 high school students	Pre and post evaluation	Connect<br>The program divides its intervention in three components: gatekeeper training for all participants;<br>discipline-specific training for professionals in 13 different disciplines (e.g., law enforcement, educators, clergy);<br>and clear, evidence-supported protocols that provide an integrated approach to guide the response of individuals who recognize a youth as being at risk for suicide.	- Significant changes in knowledge and attitudes about suicide, increased belief in the usefulness of mental health care, and reduction of stigma associated with seeking help.<br>- Adults' preparedness to help also increased significantly
47	Sandler I, Tein JY, Wolchik S, Ayers TS. The effects of the family bereavement program to reduce suicide ideation and/or attempts of parentally bereaved children six and fifteen years later. Suicide and Life-Threatening Behavior. 2016 Apr;46:S32-8.	244 children and adolescents, experienced the death of a parent between 3 and 30 months prior to beginning the program,between the ages of 8 and 16	RCT + 6 year then 15 year follow up	Family Bereavement Program (FBP) to reduce suicide ideation and/or attempts of parentally bereaved children and adolescents. is designed to enhance parenting skills, teach helpful coping methods, foster constructive communication, and create and sustain healthy parent-child relationships following the recent death of a parent or caregiver.	- The intervention effects were marginally significant at six years<br>- The intervention effects were significant at 15 years<br>-The combined 6-year and 15-year follow-up showed significant intervention effects<br>-The findings suggest that a preventive intervention, initially not designed for suicide prevention, can reduce suicidal ideation and/or attempts in parentally bereaved children
48	King KA, Strunk CM, Sorter MT. Preliminary effectiveness of Surviving the Teens® Suicide Prevention and Depression Awareness Program on adolescents' suicidality and self-efficacy in performing help-seeking behaviors. J Sch Health. 2011; 81: 581-590	966 high schoolers aged 14 to 18 yo	RCT	surviving the teens program<br>Trainings take place in the students' classrooms over the course of five days,<br>facilitating discussions about stressors that contribute to depression; teaching positive coping techniques, signs of depression and suicidal behaviors,<br>responding appropriately to troubled friends; and where to turn for help.<br>Students are taught to manage their emotions and stress reactions through problem solving, cognitive restructuring, and use of relaxation techniques. Can be used on one's self or others	- Results indicated a significant decrease in students' likelihood of currently seriously considering suicide at the 3-month follow-up compared to the pretest.<br>- Students demonstrated a significant increase in self-efficacy, both immediately after the program evaluation and at the 3-month follow-up, in recognizing signs of suicide in a friend.<br>- There was a significant increase in students' behavioral intent to inform an adult if they themselves were feeling suicidal or if a friend expressed suicidal thoughts.<br>- The majority of students reported that the program helped them learn about suicide warning signs, risk factors related to suicide and depression, effective stress coping mechanisms, steps to take if they felt suicidal, steps to take if a friend was suicidal, and improved their ability to discuss their problems with parents and friends.
49	Strunk CM, King KA, Vidourek RA, Sorter MT. Effectiveness of the Surviving the Teens® Suicide Prevention and Depression Awareness Program: An impact evaluation utilizing a comparison group. Health Education & Behavior. 2014 Dec;41(6):605-13.	high school students, 966 program group, 566 control group	RCT + pre and post evaluation	surviving the teens program	- Participants in the experiment showed significant improvement compared to the control group in their confidence in handling friends who are suicidal.<br>- There was a significant increase in the perceived importance of knowing warning signs of suicide and the intention to help among the experiment participants.<br>- Stigma-related issues concerning suicide significantly decreased among the participants.<br>- The experiment participants demonstrated a significantly greater improvement from pretest to post-evaluation compared to the control group.

Reference citation number in text	Citation of article	Population and Participants	Study Design	Targeted intervention and discription	Main quoted outcomes
50	Calear AL, McCallum SM, Christensen H, Mackinnon AJ, Nicolopoulos A, Brewer JL, Werner-Seidler A, Morse AR, Kazan D, Farrer LM, Kampel L. The Sources of Strength Australia project: A cluster randomised controlled trial of a peer-connectedness school-based program to promote help-seeking in adolescents. Journal of affective disorders. 2022 Feb 15;299:435-43.	1633 students with female majority approx 60%, from 11 to 17 yo	cluster RCT	The Sourcesof Strength program<br>a universal school-based peer leadership program developed in the United States<br>takes a social connectedness approach to improving help-seeking for suicide and general psychological distress and is designed to build socioecological protective influences across an entire school population.<br>program promotes help-seeking by changing norms about help-seeking for suicide,<br>Youth opinion leaders from diverse social cliques, including at-risk adolescents, are trained to change the norms and behaviors of their peers by conducting well-defined messaging activities with adult mentoring. 3 standard phases: (1) school and community preparation, (2) peer leader training, and (3) schoolwide messaging.	The results of the study did not support the study hypotheses, as no significant effects on help seeking were observed
51	Wyman PA, Brown CH, LoMurray M, Schmeelk-Cone K, Petrova M, Yu Q, Walsh E, Tu X, Wang W. An outcome evaluation of the Sources of Strength suicide prevention program delivered by adolescent peer leaders in high schools. American journal of public health. 2010 Sep;100(9):1653-61.	18 high school	RCT	the Sources of Strength	- Training of peer leaders with the Sources of Strength curriculum led to changes in norms across the full population of high school students after 3 months of school-wide messaging.<br>- The norms most strongly enhanced through the intervention were students' perceptions that adults in their school can provide help to suicidal students and the acceptability of seeking help from adults
52	Ohlmann C, Kwee J, Lees R. LISTENING FOR THE VOICES OF RESILIENCE: A GROUP OF ADOLESCENTS'EXPERIENCES WITH A SUICIDE PREVENTION EDUCATION PROGRAM. International Journal of Child, Youth and Family Studies. 2014 Jan 20;5(1):24-46.	5 highs school students aged 15 to 18 identified by school staff as being at tisk for self harm or suicide	Qualitative method of the Listening Guide	The Listening Guide is a qualitative, relational, voice-centered, feminist methodology used to analyze interviews<br>It is a method that intentionally brings a strong relational component to the analysis<br>It is not a specific intervention for suicide prevention but rather a qualitative research method used to analyze interviews and narratives	- Overarching themes: voices of vulnerability and voices of resilience<br>- Through the connection in the Alive group, the participants came to know themselves more deeply, and they became more confident in trusting their own intuition. Becoming more familiar with who they are and who they want to be, they were better able to maintain a healthy emotional state.
53	Lindow JC, Hughes JL, South C, Gutierrez L, Bannister E, Trivedi MH, Byerly MJ. Feasibility and acceptability of the youth aware of mental health (YAM) intervention in US adolescents. Archives of suicide research. 2020 Apr 2;24(2):269-84.	436 students with a mean age of 14 yo	Survey	Youth Aware of Mental health (YAM) a school-based program for young people aged 13 to 17,<br>aims to raise awareness about mental health and promote positive mental health practices<br>enhance the skills and emotional resiliency needed to deal with adverse life events, stress, and suicidal behavior,	- The YAM group experienced a significant reduction in suicidality over 1 year.<br>- This reduction included 55% fewer incident suicide attempts and 50% fewer cases of severe suicidal ideation compared to the control group.<br>- The study concluded that YAM is feasible to implement in US schools

Reference citation number in text	Citation of article	Population and Participants	Study Design	Targeted intervention and discription	Main quoted outcomes
54	Godoy Garraza L, Kuiper N, Goldston D, McKeon R, Walrath C. Long-term impact of the Garrett Lee Smith Youth Suicide Prevention Program on youth suicide mortality, 2006–2015. Journal of child psychology and psychiatry. 2019 Oct;60(10):1142-7.	1126 counties exposed, 969 counties unexposed	Prospective Cohort Study	Garrett Lee Smith Program: govenrment and cummunity effort to lower suicide rates.<br>These programs support the development and implementation of suicide prevention and early intervention strategies focused on youth aged 10–24. GLS grantees utilize funding for a variety of suicide prevention interventions, including gatekeeper trainings; outreach and awareness strategies; early identification screening programs; partnership development for early intervention, linkages to community providers and appropriate treatment; care transitions; culturally based prevention activities; and means restriction	- The implementation of GLS activities was associated with a decrease in youth suicide mortality rates. In the year following the implementation<br>- Two years after the implementation of GLS activities, youth suicide mortality rates were estimated to be 1.1 per 100,000 youths lower than they would have been without the program.<br>The impact of GLS activities appears to strengthen with longer exposure. In the absence of the program, it was expected that there would be 3.32 fewer deaths per 100,000 youths over a 4-year period<br>- Similarly, over a 7-year period, the absence of the program would have resulted in 13.3 fewer deaths per 100,000 youths
55	Walrath C, Garraza LG, Reid H, Goldston DB, McKeon R. Impact of the Garrett Lee Smith youth suicide prevention program on suicide mortality. American Journal of Public Health. 2015 May;105(5):986-93.		Non randomized quasi experimental	Garrett Lee Smith (GLS) program<br>comparing counties with and without GLS tranings	- Counties implementing GLS training had significantly lower suicide rates among the population aged 10 to 24 years the year after GLS training<br>- Simultaneously, we found no significant difference in terms of adult suicide mortality rates or nonsuicide youth mortality the year after the implementation
56	Chamlong Disayavanish M, Primprao Disayavanish M, Tala Thammaroj M, Surut Jianmongkol M, Kimaporn Kamanarong M, Somjit Prueksaritanond M. A Buddhist approach to suicide prevention. J Med Assoc Thail. 2007;90(8):1680-8.	thailand's buddist young people	Case study	Spiritual beliefs coinciding with many well-documented primary prevention strategies for suicide	Approach 3: Providing monks with general knowledge and understanding about suicide and life after death.<br>Approach 4: Early identification of mental disorders, individuals at risk of suicide, and ensuring prompt referral to appropriate mental health professionals.<br>Approach 5: Implementation of measures to control access to instruments of suicide.<br>Approach 6: Implementation of measures to control alcohol and drug abuse.<br>Approach 8: Promoting responsible media reporting on suicide-related matters.<br>Approach 9: Encouraging the practice of meditation.
57	Vendetti T, Hill J. Linking public schools and community mental health services: A model for youth suicide prevention. Rhode Island Medical Journal. 2018 May 1;101(4):36-8.	328 elemantary school students	Pre and post evaluation + follow up	Suicide Prevention Initiative (SPI). SPI is a youth suicide prevention referral system that links public elementary, middle and high schools with mental health services. T<br>The program supposedly diverts at-risk students who express suicidal ideation and/or non-suicidal self-harm from unnecessary Emergency Department (ED) visits by connecting the student to local mental health services with follow-up support.	- Among the referred students, approximately 62.0% were girls.<br>- The majority of parents (approximately 75%) reported that their child was actively engaged in therapy and experiencing improvements.<br>- Approximately 15% of parents believed that therapy was unnecessary for their child and expressed either "no concerns" or frustration towards the school and mental health systems for stigmatizing their child.

Reference citation number in text	Citation of article	Population and Participants	Study Design	Targeted intervention and discription	Main quoted outcomes
58	Crosby Budinger M, Cwik MF, Riddle MA. Awareness, attitudes, and use of crisis hotlines among youth at-risk for suicide. Suicide and Life-Threatening Behavior. 2015 Apr;45(2):192-8.	168 youth aged 10 to 17 yo	Survey	Utilization of crisis hotline in Maryland	- The primary barrier identified by these youth was the stigma associated with seeking help, which acted as a deterrent
59	Inscoe AB, Donisch K, Cheek S, Stokes C, Goldston DB, Asarnow JR. Trauma-informed care for youth suicide prevention: A qualitative analysis of caregivers' perspectives. Psychological trauma: theory, research, practice, and policy. 2022 May;14(4):653.	13 caregivers of youth with trauma history and STB	Grounded Theory	Trauma-informed care shifts the focus from "What's wrong with you?" to "What happened to you?"<br>A trauma-informed approach to care acknowledges that health care organizations and care teams need to have a complete picture of a patient's life situation — past and present — in order to provide effective health care services with a healing orientation<br>Done with cargiver of youth, to identify postive and negative clincial care in youth with trauma history	- Parent involvement was identified as one of the most effective interventions for addressing youth suicidal thoughts and behaviors.<br>- Clinician characteristics such as authenticity, genuineness, empathy, warmth, nonjudgmental stance, and validation were recognized as crucial in facilitating positive outcomes.<br>- Given the high prevalence of childhood traumatic experiences, particularly among youth in acute suicidal crisis, it is crucial for healthcare providers to identify signs of traumatic stress and respond appropriately to prevent unintended re-traumatization.<br>- The interviews also highlighted the significant difficulties parents faced in navigating the mental health system and finding suitable care for their children
60	Chen SS, Lam TP, Lam KF, Lo TL, Chao DV, Mak KY, Lam EW, Tang WS, Chan HY, Yip PS. Motivations for online expression, willingness of online help-seeking, and the risk of suicide among Hong Kong youths: a mixed-methods study. Cyberpsychology, Behavior, and Social Networking. 2022 Jun 1;25(6):384-91.	students aged 10 to 12 and 15 to 19	Exploratory sequential mixed-methods	Evaluating the efficacy of online self-expression through interviews and surveys.	- Online platforms are primarily used for self-expression rather than seeking help, and no participants have attempted to seek help online.<br>- Survey responses indicated that online expression is used for sharing, self-expression, seeking attention, social interactions, emotional ventilation, one-way expression, and trusting the platform, but there is reluctance to seek help.
61	Chen SS, Lam TP, Lam KF, Lo TL, Chao DV, Mak KY, Lam EW, Tang WS, Chan HY, Yip PS. The use of close friends on Instagram, help-seeking willingness, and suicidality among Hong Kong youth: exploratory sequential mixed methods study. Journal of medical internet research. 2022 Oct 12;24(10):e37695.	31 in focus group, 12 in individual interviews, 3 for both all aged 5 to 18	Exploratory sequential mixed-methods	Exploring the association between *Close friends list* on Instagram and the online and offline help-seeking behavior	- Close Friends offers a secure online platform for private communication and self-expression.<br>- The platform creates an environment that encourages at-risk adolescents to be more authentic in their self-disclosure of distress and exploration of identity.
62	Olejniczak D, Jabłkowska-Górecka K, Panczyk M, Gotlib J, Walewska-Zielecka B. Analysis of adolescents' opinions on suicide prevention. Psychiatr. Pol. 2019;53(2):359-70.	1,439 respondents participated in the study – 821 girls (57.1%) and 592 boys (41.1%)	Survey	Awarness and implication of social actors in suicide prevention	Family implication was considered 70% effective topping all other social actors like church and police and shool

Reference citation number in text	Citation of article	Population and Participants	Study Design	Targeted intervention and discription	Main quoted outcomes
63	Robinson J, Hill NT, Thorn P, Battersby R, Teh Z, Reavley NJ, Pirkis J, Lamblin M, Rice S, Skehan J. The# chatsafe project. Developing guidelines to help young people communicate safely about suicide on social media: A Delphi study. PLoS One. 2018 Nov 15;13(11):e0206584.		Tool development + evaluation	#chatsafe guidelines:Guidline of 173 items: 1)Before you post anything online about suicide; 2)Sharing your own thoughts, feelings, orexperience with suicidal behaviour online; 3)Communicating about someone you know who isaffected by suicidal thoughts, feelings orbehaviours; 4) Responding tosomeone who may be suicidal; 5)Memorial websites, pages and closed groups tohonour the deceased.	*To be tested for effectiveness*
64	Robinson J, Teh Z, Lamblin M, Hill NT, La Sala L, Thorn P. Globalization of the# chatsafe guidelines: Using social media for youth suicide prevention. Early intervention in psychiatry. 2021 Oct;15(5):1409-13.	48 online participants	Tool development + evaluation	#chatsafe guidelines	- The international guidelines were downloaded approximately 4,100 times, indicating their reach and utilization.<br>- The social media content related to the guidelines has reached over one million young people across the partner regions, further disseminating the information.
65	Hubbard M. Suicide Prevention: Reducing Self-Harm in Adolescents using the Smart Phone App “Calm Harm”. Journal of Medical & Clinical Nursing. SRC/JMCN-160. DOI: doi. org/10.47363/JMCN/2022 (3). 2022;148:2-7.	10 initial participant, 8 remained, 15-17yo with a past history of self-harm thoughts or behaviors.	Pre and post evaluation	Questions were used to assess thoughts about suicide and self-injuring behaviors within the four weeks prior to the pretest questionnaire and during the four weeks while using the Calm Harm app using the post-study questionnaire.	Participants were generally pleased with the app and used it during specific times.
66	O'Grady C, Melia R, Bogue J, O'Sullivan M, Young K, Duggan J. A mobile health approach for improving outcomes in suicide prevention (SafePlan). Journal of medical internet research. 2020 Jul 30;22(7):e17481.	usability testing done by 18 students 14 to 16 yo	Tool development + survey evaluation	A fully functional mobile app, known as the SafePlan app, was developed and tested with the input of clinicians through an iterative design process. The app’s core function is to provide an interactive safety plan to support users with suicidal thoughts or behaviors as an adjunct to face-to-face therapy	An mHealth intervention technology that has the potential to increase accessibility to this type of mental health service for the target population
67	Johnson LA, Parsons ME. Adolescent suicide prevention in a school setting: Use of a gatekeeper program. NASN school nurse. 2012 Nov;27(6):312-7.	approximately 3,000 students and 400 staff members.	Pre and post evaluation + follow up	QPR Suicide Prevention Program<br>(paid program)	- One staff member implemented the QPR protocol using the question, persuasion, and referral technique to manage a potentially suicidal student, as identified in the e-mail questionnaire response.<br>- The remaining 35 staff members did not implement the QPR Program, stating that they had not encountered any students whom they believed were suicidal.<br>- It is important to note that no suicide attempts were reported within the schools during the specified timeframe

Reference citation number in text	Citation of article	Population and Participants	Study Design	Targeted intervention and discription	Main quoted outcomes
68	Jenner E, Jenner LW, Matthews-Sterling M, Butts JK, Williams TE. Awareness effects of a youth suicide prevention media campaign in Louisiana. Suicide and life-threatening behavior. 2010 Aug;40(4):394-406.	Approx 24 000 calls in 47 months from louisiana youth	Quasi experimental	LPYSP media campaigns, The implied theory here is that if the target audience is alerted to the existence of the hotline through direct or indirect exposure, then people will know where to turn for help and act on that knowledge.<br>Follow up of the use of the lifeline hotline from 2005 to 2008 with two campaign for lifeline hotline launched in between	- In early 2005, the monthly call counts were consistently below 100. However, by 2008, the number of calls increased approximately tenfold.<br>- The average monthly call count in 2005 was 262. Subsequently, it climbed to 474, 563, and reached a peak of 774 over the next three years.<br>- Notably, there was a significant spike in call volumes in September 2005, immediately following Hurricane Katrina. The highest number of calls, 920, was recorded in October of that year.<br>- In 2006, the call volumes did not return to pre-September levels, nor did they exhibit the same spikes observed in 2005. It is reasonable to infer that the trauma associated with Hurricane Katrina may have played a role in this pattern.
69	Freedenthal S. Adolescent help-seeking and the yellow ribbon suicide prevention program: An evaluation. Suicide and life-threatening behavior. 2010 Dec;40(6):628-39.	1600 control students, 870 in expirment group	Pre and post evaluation + follow up	Yellow Ribbon program<br>Increase help seeking behavior<br>These included a 60-minute student leadership train ing program for about 15 students selected by school counselors; a 1.5 hour staff training teachers, counselors, administrators, and all other school staff; and a 50-minute schoolwide assembly.	No particular change in help-seeking behavior
70	Chagnon F, Houle J, Marcoux I, Renaud J. Control-group study of an intervention training program for youth suicide prevention. Suicide and Life-Threatening Behavior. 2007 Apr;37(2):135-44.	43 helpers who received the training were compared with 28 helpers who did not	Pre and post evaluation + follow up	A training program has already been implemented at 'Suicide Action MontReal' for helpers involved with youth. The program is conducted once a week for three weeks and aims to train helpers in recognizing warning signs, identifying alternative means of solving youth problems other than suicidal behavior, and referring them to appropriate resources	- A 15% increase in knowledge and improved skills was observed.<br>- The effects of the intervention were found to be sustained for at least 6 months.
71	Bagatelas PH, Hunt JI. Assessing the quality of patient responses to a psychosocial intervention implemented on an adolescent psychiatric inpatient unit: devising the safety plan quality metric. Rhode Island Medical Journal. 2022 May 1;105(4):22-5.	145 participants hospitalized on an adolescent psychiatric unit	Non randomized quasi experimental	The Safety Planning Intervention (SPI) helps patients use coping strategies when in a suicidal crisis.Typically, a safety plan is developed collaboratively by the psychologist and client while the client is not in crisis.<br>- correlation betwee quality of SPI and readmission to an inpatient psychiatric hospital	Implementing effective SPI measures may contribute to reducing the likelihood of individuals being readmitted to psychiatric hospitals for mental health-related concerns.
72	Xavier A, Otero P, Blanco V, Vázquez FL. Efficacy of a problem-solving intervention for the indicated prevention of suicidal risk in young Brazilians: Randomized controlled trial. Suicide and Life-Threatening Behavior. 2019 Dec;49(6):1746-61.	100 participants (60% women,) 15 and 19 years old, having high suicidal orientation and high depressive symptoms	RCT	problem solving therapy cognitive–behavioral intervention geared to improve an individual's ability to cope with stressful life experiences.<br>evaluate the efficacy of a psychological intervention	- The problem-solving group showed a significant reduction in suicidal orientation compared to the control group, and this reduction was maintained at the 6-month follow-up, with a large effect size.<br>- There were no significant differences between the problem-solving group and the control group regarding suicide and suicide attempts

Reference citation number in text	Citation of article	Population and Participants	Study Design	Targeted intervention and discription	Main quoted outcomes
73	Grummitt LR, Debenham J, Kelly E, Barrett EL, Champion K, Conrod P, Teesson M, Newton N. Selective personality-targeted prevention of suicidal ideation in young adolescents: post hoc analysis of data collected in a cluster randomised controlled trial. Medical journal of Australia. 2022 Jun 6;216(10):525-9.	755 control groupp, 881 intervention. Participants are 13yo students	RCT + 6 months, 1, 2, and 3 year follow up	preventure: a selective personnality targetted intervention.<br>- The sessions targets specific high-risk personality traits: negative thinking; anxiety sensitivity; impulsivity; and sensation seeking<br>- The sessions are intended to educate participants about their personality types and to identify and challenge personality-specific cognitive distortions that lead to problematic behaviours.<br>- The first session used psycho-education to help students understand the characteristics of their personality style and to explore unhelpful coping behaviours specific to their personality risk group.<br>- The second session encouraged students to challenge personality-specific thoughts that elicit problematic emotional and behavioural reactions.	- Across the three-years of follow-up, the annual reduction in the odds of suicidal ideation was greater for the intervention group than the control group<br>- Over the three-year follow-up period, the intervention group exhibited a greater annual reduction in the odds of suicidal ideation compared to the control group.
74	Mouchabac S, Leray P, Adrien V, Gollier-Briant F, Bonnot O. Prevention of suicidal relapses in adolescents with a smartphone application: Bayesian network analysis of a preclinical trial using in silico patient simulations. Journal of Medical Internet Research. 2021 Sep 30;23(9):e24560.	computer based "in silico"	Tool development + survey evaluation	In the context of suicide Preventure, Bayesian network analysis could be used to model the relationships between personality traits, coping skills, and substance use. The analysis could help identify which personality traits are most strongly associated with substance use and which coping skills are most effective in reducing substance use for individuals with different personality profiles	The pattern of advice converges to a solution that provides priority advice without requiring interventions from caregiver
75	MacDonald, S., Sampson, C., Biddle, L., Yeon Kwak, S., Scourfield, J., & Evans, R. (2020). Theorising health professionals' prevention and management practices with children and young people experiencing self-harm: A qualitative hospital-based case study.	14 healthcare professionals	Case study	To understand the practices of health professionals in the hospital setting and affiliated care settings when treating children and young people who present following self-harm	The system faces challenges in having or finding expertise to treat self-harm effectively.
76	Kennard BD, Goldstein T, Foxwell AA, McMakin DL, Wolfe K, Biernesser C, Moorehead A, Douaihy A, Zullo L, Wentroble E, Owen V. As Safe as Possible (ASAP): a brief app-supported inpatient intervention to prevent postdischarge suicidal behavior in hospitalized, suicidal adolescents. American journal of psychiatry. 2018 Sep 1;175(9):864-72.	66 adolescents hospitalized for suicidal ideation (N=26) or a recent suicide attempt (N=40)	Pilot study	As Safe as Possible (ASAP), As Safe as Possible (ASAP) is a brief app-supported inpatient intervention designed to prevent post-discharge suicidal behavior in hospitalized, suicidal adolescents<br>The intervention program focuses on emotion regulation and safety planning and is delivered on the inpatient unit over a 3-hour period<br>The BRITE app is used to support the intervention and prompts participants to rate their level of emotional distress on a daily basis and provides personalized strategies for emotion regulation and safety planning	-The study found no treatment effects on suicidal ideation.<br>- Participants expressed high satisfaction with both the intervention and the app
77	Czyz EK, King CA, Prouty D, Micol VJ, Walton M, Nahum-Shani I. Adaptive intervention for prevention of adolescent suicidal behavior after hospitalization: a pilot sequential multiple assignment randomized trial. Journal of child psychology and psychiatry. 2021 Aug;62(8):1019-31.	Adolescent inpatients (N = 80; ages 13–17; 67.5% female	Pilot Study - RCT	The MI-SafeCope intervention includes three components: (1) MI-enhanced safety plan delivered during hospitalization (encompassing individual and family meetings); (2) post-discharge booster call; and (3) post-discharge daily text message boosters.<br>Phase 1 delivered during hospitalization, alone or in combination with postdischarge text-based support (Texts). Two weeks after discharge, participants were re-randomized in Phase 2 to added telephone booster calls or to no calls.<br>4 group comparison all receinving a different form of intervention.<br>Compared baselin, during interventon, 1 and 3 month after intervention	- In Phase 1, there were no differences in the decrease of suicidal ideation severity between adolescents assigned to MISP and MI-SP + Texts during the 1- and 3-month follow-up.<br>- In Phase 2, there were no differences in the decrease of suicidal ideation severity between adolescents assigned to booster calls and those with no calls.<br>- MI-SP + Texts showed a lower risk of suicide attempts compared to MI-SP, although the difference was not statistically significant.

Reference citation number in text	Citation of article	Population and Participants	Study Design	Targeted intervention and discription	Main quoted outcomes
78	Mancinelli-Hough K, Lucas Breda K, Karl C, Wentland BA. Don't Ask, Won't Tell: Suicide Screening in the Pediatric Perioperative Setting. Comprehensive child and adolescent nursing. 2022 Oct 2;45(4):395-402.	22 perioperative nurses	Pre and post evaluation	educational component about universal suicide screening on children and adolescents.	After the education program, all nurse participants agreed or strongly agreed that it was within their scope to screen for SI and SH
79	Lindquist-Grantz R, Abraczinskas M. Using youth participatory action research as a health intervention in community settings. Health promotion practice. 2020 Jul;21(4):573-81.		Tool development + evaluation	Youth participatory action research (YPAR)<br>a cyclical process of learning and action in which young people are trained to conduct systematic research to improve their lives, their communities, and the institutions intended to serve them<br>engages young people in research on issues that matter to them and builds their capacity to take action within their own schools and communities<br>not specific for suicide prevention	- We have initiated a set of best practices for using Youth Participatory Action Research (YPAR) in the health sciences.<br>- However, it is important to note that these best practices do not encompass all aspects of using YPAR as a health intervention in community-based settings
80	Cohen R, Rifkin-Zybutz R, Moran P, Biddle L. Web-based support services to help prevent suicide in young people and students: A mixed-methods, user-informed review of characteristics and effective elements. Health & Social Care in the Community. 2022 May 6.	students aged 16–25 years	A sequential cross-sectional mixed-methods	This study mapped online help provision for YP and students experiencing acute mental or suicidal crises.	- General Practitioners (GPs) observed that young people (YP) are less likely to resist mental health support if it is delivered in an age-sensitive manner.<br>- Young people praised the simplicity of the information provided on a service website during interviews.<br>- Young people generally desire immediate information and quick solutions when seeking help.<br>- Young people expressed a preference for written communication, such as text and webchat, over verbal methods like phone calls when reaching out for help during a crisis.
81	Totura CM, Kutash K, Labouliere CD, Karver MS. Evaluating active parental consent procedures for school programming: addressing the sensitive topic of suicide prevention. Journal of school health. 2017 Feb;87(2):114-20.		Tool development + evaluation	Evaluating parents response and participation consent in school based intervention studies<br>Five active consent methods (in-person, students taking forms home, mailing, mailing preceded by primers, mailing followed by reminder calls) were compared against passive consent procedures to evaluate recruitment success,	- In-person methods yielded higher rates of response and participation than less interpersonal methods like mailing or students taking consents home.<br>- The inclusion of mailed primers before or reminder calls after consent forms were sent increased response rates, but did not have a significant impact on participation rates.<br>- Students taking consents home had the lowest rates of response and participation compared to other methods.
82	Kutcher S, Wei Y, Behzadi P. School-and community-based youth suicide prevention interventions: hot idea, hot air, or sham?. The Canadian Journal of Psychiatry. 2017 Jun;62(6):381-7.	6 studies	Systematic Review	Investigate the quality of available evidence of the effectiveness of SafeTALK<br>a suicide prevention program that aims to increase suicide alertness and help individuals become more comfortable and confident in responding to individuals who may be at risk of suicide<br>a half-day face-to-face training program that includes powerful presentations, audiovisuals, and skills practice<br>teaches individuals to recognize the signs of suicide, engage with the person at risk, and connect them with resources that can help	- All 6 studies fell into the OJP "insufficient evidence" category<br>- In none of the above popularly applied suicide prevention programmes, such as SOS, Yellow Ribbon, and SafeTALK, was any sufficient evidence of effectiveness or of safety found

Reference citation number in text	Citation of article	Population and Participants	Study Design	Targeted intervention and discription	Main quoted outcomes
83	Rani MR, Krupanidhi S. A study to assess the effectiveness of structured teaching programme on suicide prevention among adolescents at selected higher secondary schools in Krishna District, Andhra Pradesh. IJAR. 2020;6(9):219-23.	250 adolescent	Pre and post evaluation	A structured teaching program on suicide prevention designed to educate individuals on the risk factors and preventive measures for suicidal behavior<br>(unlcear program execution and implementation)	- Structured teaching programme on suicide prevention was highly effective.<br>- There was significant association between the demographic variables and post-test level of knowledge on suicide prevention.
84	Kaur G, Sathish R. Effectiveness of Information Education and Communication on Knowledge Regarding Suicide and its Prevention among Adolescents in Selected Schools of Panipat. International Journal of Nursing Education. 2020 Jan 31;12(1):11-4.	60 students	RCT + pre and post evaluation	Information Education and Communication (IEC) was given with the help of AV aids like PowerPoint and LCD.<br>It involves providing information and education to individuals and communities to increase awareness and understanding of suicide risk factors, warning signs, and preventive measures<br>Can be implemented through various channels such as social media, websites, brochures, posters, and public service announcements	Significant effect on gaining knowledge of adolescents in the selected setting.
85	Labouliere CD, Tarquini SJ, Totura CM, Kutash K, Karver MS. Revisiting the concept of knowledge: how much is learned by students participating in suicide prevention gatekeeper training?. Crisis: The Journal of Crisis Intervention and Suicide Prevention. 2015;36(4):274.	demographic analyses were based on 852 participants, whereas knowledge analyses include all 1,365 participants	Pre and Post survey + focus groups	The Jason Foundation "A Promise for Tomorrow" gatekeeper prevention program<br>promotes awareness of the problem of youth suicide,<br>provides students with the knowledge and resources to interact with at-risk youth,<br>develops positive attitudes toward referring atrisk peers.<br>The training sessions were conducted by teachers or counselors during health classes, were overseen by school mental health personnel	- Unlike in studies using forced-choice assessment, students' baseline knowledge was markedly low using free-recall questions and, despite making significant improvement from pretraining levels, posttraining knowledge barely approached passable levels.
86	Totura CM, Labouliere CD, Gryglewicz K, Karver MS. The role of youth trainee–trainer alliance and involvement in school-based prevention: A moderated-mediation model of student gatekeeper suicide prevention training. Administration and Policy in Mental Health and Mental Health Services Research. 2019 Mar 15;46:209-19.	418 adolescents students	Moderated-mediation design	The Jason foundation A promise for tomorrow<br>The relationship between preand post-training intentions to refer are mediated by alliance and the relationship between alliance and post-training intentions is moderated by student involvement in training	Having intentions to act does not suggest that a sufficient amount of prevention skill development occurred to act effectively.
87	Heinz A, Catunda C, van Duin C, Willems H. Suicide prevention: Using the number of health complaints as an indirect alternative for screening suicidal adolescents. Journal of affective disorders. 2020 Jan 1;260:61-6.	5262 adolescents between 12 and 18 yo	Tool development + survey evaluation	HBSC 2014 survey, including the HBSC-SCL items and suicidal ideation and behavior questions.<br>The HBSC-SCL (Health Behaviour in School-aged Children Symptom Checklist) is a screening tool for suicide prevention in youth<br>Two earlier studies have shown that HBSC-SCL consists of two sub-dimensions that are highly correlated: somatic complaints and psychological complaint, can also be used as a unidimensional measure of psychosomatic health	- Cronbach's alpha analysis is acceptable with α = 0.84 and the exclusion of any item would lower this value<br>- All correlations between the eight HBSC-SCL items and the four SIB items are statistically significant<br>- All areas under the ROC curves are significantly greater than 0.5, meaning that each cut-off value gives results that are better than guesswork<br>- he HBSC-SCL scale is reliable and has good internal consistency which is also in line with previous research

Reference citation number in text	Citation of article	Population and Participants	Study Design	Targeted intervention and discription	Main quoted outcomes
88	Breux P, Boccio DE. Improving schools' readiness for involvement in suicide prevention: An evaluation of the creating suicide safety in schools (CSSS) workshop. International journal of environmental research and public health. 2019 Jun;16(12):2165.	Participants (N = 562) consisted predominantly of school-based mental health professionals	Tool development + pre and posttest + survey evaluation + follow up	the Creating Suicide Safety in Schools (CSSS) workshop, on the readiness of school personnel to improve their schools' suicide-related policies and procedures	- The increases observed at post-test were relatively modest and not likely to be clinically meaningful.
89	Bailey E, Spittal MJ, Pirkis J, Gould M, Robinson J. Universal suicide prevention in young people. Crisis. 2017 Jul 27.	129 students from the three main high schools	Tool development + survey follow up	Evaluate the efficacy and acceptability of the safeTALK program for secondary school students and determine whether it is associated with any iatrogenic effects.<br>Participants were assessed immediately before and immediately after the training using a purpose-designed survey. Follow-up questionnaires were administered online four weeks after completion of the training.	- Participants demonstrated increases in knowledge about suicide, confidence in talking about issues related to suicide, willingness to talk about suicide, and likelihood of seeking help for suicidal thoughts.<br>- These increases were evident at Time 2 and were generally sustained over the follow-up period.
90	Vaughn LM, Sunny CE, Lindquist-Grantz R, King C, Brent D, Boyd S, Grupp-Phelan J. Successful suicide screening in the pediatric emergency department: youth, parent, researcher, and clinician perspectives. Archives of suicide research. 2020 Feb 3;24(sup1):124-41.	149 included a purposeful sample of relevant stakeholders (clinicians and social service providers 11% researchers involved with suicide screening, awareness, and prevention, 11% youth members of the YCSP, 13% parents of YCSP youth or who have been to the pediatric ED with their teenager).	Mixed method design by using concept mapping	mixed method design by using concept mapping (CM) The method produces a visual concept map that can easily be understood and used by stakeholders to conceptualize a complex problem and develop solutions for addressing it<br>Most concept maps highlight one main idea and depict subtopics as boxes or circles connected with lines, arrows, and (or) linking words<br>Concept mapping encourages to zoom out and start with a broader perspective before diving into the details	Members of the different stakeholder groups in this study did not agree completely on the strategies that were most important for suicide screening or on the relative feasibility of various approaches
91	Asarnow JR, Hughes JL, Babeva KN, Sugar CA. Cognitive-behavioral family treatment for suicide attempt prevention: a randomized controlled trial. Journal of the American Academy of Child & Adolescent Psychiatry. 2017 Jun 1;56(6):506-14.	42 youths in the randomized intent-to-treat sample: SAFETY (n=20); E-TAU (n=22)	RCT	SAFETY or treatment as usual enhanced by parent education and support accessing community treatment (E-TAU)	Survival analyses indicated a significantly higher probability of survival without a suicide attempt by the 3-month follow-up point among SAFETY youths and for the overall survival curves
92	Calear AL, Christensen H, Freeman A, Fenton K, Busby Grant J, Van Spijker B, Donker T. A systematic review of psychosocial suicide prevention interventions for youth. European child & adolescent psychiatry. 2016 May;25:467-82.	29 articles included	Systematic Review	Counselors Care, Assess, Respond, Empower (C-CARE), Coping and Support Training (CAST) and/or Parents Care, Assess, Respond, Empower (P-CARE) programs	just over half of the programs identified in the review reported significant effects on suicidal ideation, suicide attempts or deliberate self-harm
